# Butyrate driven raft disruption trots off enteric pathogen invasion: possible mechanism of colonization resistance

**DOI:** 10.1186/s13099-023-00545-0

**Published:** 2023-04-21

**Authors:** Oishika Das, Aaheli Masid, Mainak Chakraborty, Animesh Gope, Shanta Dutta, Moumita Bhaumik

**Affiliations:** ICMR-National Institute of Cholera and Enteric Diseases, P-33 C.I. T Road, Beleghata, Kolkata, West Bengal 700010 India

**Keywords:** Butyrate, Cholesterol, Lipid raft, Pathogen invasion

## Abstract

The gut microbiome derived short chain fatty acids perform multitude of functions to maintain gut homeostasis. Here we studied how butyrate stymie enteric bacterial invasion in cell using a simplistic binary model. The surface of the mammalian cells is enriched with microdomains rich in cholesterol that are known as rafts and act as entry points for pathogens. We showed that sodium butyrate treated RAW264.7 cells displayed reduced membrane cholesterol and less cholera-toxin B binding coupled with increased membrane fluidity compared to untreated cells indicating that reduced membrane cholesterol caused disruption of lipid rafts. The implication of such cellular biophysical changes on the invasion of enteric pathogenic bacteria was assessed. Our study showed, in comparison to untreated cells, butyrate-treated cells significantly reduced the invasion of *Shigella* and *Salmonella*, and these effects were found to be reversed by liposomal cholesterol treatment, increasing the likelihood that the rafts' function against bacterial invasion. The credence of ex vivo studies found to be in concordance in butyrate fed mouse model as evident from the significant drift towards a protective phenotype against virulent enteric pathogen invasion as compared to untreated mice. To produce a cytokine balance towards anti-inflammation, butyrate-treated mice produced more of the gut tissue anti-inflammatory cytokine IL-10 and less of the pro-inflammatory cytokines TNF-α, IL-6, and IFN-γ. In histological studies of *Shigella* infected gut revealed a startling observation where number of neutrophils infiltration was noted which was correlated with the pathology and was essentially reversed by butyrate treatment. Our results ratchet up a new dimension of our understanding how butyrate imparts resistance to pathogen invasion in the gut.

**Supplementary Information:**

The online version contains supplementary material available at 10.1186/s13099-023-00545-0.

## Introduction

The community of bacteria that lines the intestinal tract is an epitome of symbiotic relation with the host [1]. The intestine of mammals is colonized by a complex group of bacteria. The commensal gut bacteria stymies pathogen colonization by exploiting array of mechanisms including ecological competition for nutrients, active antagonism by secretion of antimicrobial peptides and bactericidins and metabolite mediated inhibition [2]. The gut microbiota has been linked to restoring biological functions of the host including metabolism, gut barrier function and immune development [3]. Furthermore, the commensal microbes resist growth of opportunistic bacteria protecting the host from pathogen infection, phenomena called “colonization resistance” [4]. The pioneering work demonstrating dramatic increase in susceptibility to *Salmonella enteritidis* infection in antibiotic mediated disruption of gut bacteria in mice opened critical window to address the problem with centrality [5]. Metabolites produced by the gut microbes especially short chain fatty acids (SCFA) constituting mainly aceatate, propionate and butyrate plays an important role in resisting pathogen from colonization [6]. A link between SCFAs and *Clostridium difficile* infection was first discovered in 1994 that found pigs fed a high-fiber diet were less susceptible to *C. difficile* infection [7]. Later, copious reports demonstrated that loss of SCFA production correlated with susceptibility to enteric infection [8], [9]. SCFA is shown to imprint antimicrobial properties in macrophages even in the absence of increased inflammatory cytokine response [10]. SCFA also provide an important resistance mechanism against pathogen by exerting toxic acid stress [8] as demonstrated in vitro that the toxicity was attributable to the non-ionized forms of these acids, which exist more prominently at low pH [8, 11] SCFA also plays a critical role in maintaining the limited availability of oxygen and nitrate leading to decrease *Salmonella* infection [12]. Considering SCFAs regulate multiple metabolic pathways of the host [13], the contribution of SCFA mediated antagonism to resist colonization in light of host cell function needs to be evaluated.

Earlier we have shown that butyrate but not aceatate or propionate regulates cholesterol homeostasis exploiting a probable axis “AUF-1-Dicer1-miR122-cholesterol” [14]. Cholesterol is an important component of the cell membrane, modulating a plethora of biophysical properties of the membrane. A cholesterol-rich station in the membrane known as a lipid raft is formed when cholesterol is present in membranes with pre-determined membrane dynamics [15]. These lipid rafts are intended to serve as a gateway for the entry of numerous pathogens [16]. Pathogens can elude the immune system in an innovative way by using membrane microdomains [16]. Some pathogens have acquired strategies to thwart innate and adaptive immune responses by co-opting raft-associated pathways. Most intracellular pathogens tactically exploit lipid rafts for entering the host cell. *Salmonella* and *Shigella* have a common requirement for a T3SS (type III secretion system), which is a multicomponent molecular syringe that allows the translocation of so called effector proteins from bacterial cytoplasm, through the inner and outer bacterial membrane, as well as the host plasma membrane, directly into cytoplasm [17]. It has effector proteins called SipB and SipC for *Salmonella* [18] and IpaB and IpaC for *Shigella* [19] that must come into contact with the host cell in order to activate the system*.* The binding of SipB/IpaB to host cells requires cholesterol in the membranes on the downstream of the T3SS activation [20]. The pathogenic vesicles of Enterotoxigenic *E. coli* fuses to the cell membrane in the cholesterol rich domain of the host cell membrane [21]. Cholesterol depleting agents reduces internalization of *Helicobacter pylori* in gut epithelial cells [22]. Hole forming bacteria like *Vibrio cholerae* prefers to create holes in the lipid rafts as rafts can sustain the holes for longer time [23].

Armed with credible studies we aim to study if butyrate that reduces cholesterol biosynthesis can disrupt lipid rafts and prevents pathogen invasion. We studied butyrate treatment reduces the cholesterol content and alters the physical properties of cell membrane. Here we performed fluorescence polarization using Laurdan probe for measuring the fluidity in cell membranes with or without butyrate treatment. Employing gentamycin protection assay we have shown that butyrate prevents invasion of *Shigella* and *Salmonella* in macrophages. Further leverage on reduced pathogen invasion on butyrate treatment was due to lack of membrane cholesterol was stemmed from restoration of cholesterol in membranes by liposomal delivery which showed reversal of butyrate effect. Our observation in cell line also resonates in mice model. By harmonising narratives from our experimental studies, we showed that gut microbial butyrate decreases membrane cholesterol and disrupts lipid rafts resulted in decrease in pathogen invasion, a "critical" denominator for pathogen resistance. Our study provides additional unique forces of pathogen resistance in gut.

## Materials and methods

### Reagents and chemicals

Chow diet (Harlan Teklad LM-485) was purchased from ICMR-NIN, Hyderabad, India. Dulbecco′s modified Eagle′s medium (DMEM) and foetal calf serum (FCS) were purchased from GIBCO (Waltham, MA, USA). Gentamycin, BCA protein assay kit, apoptosis kit, LDH kit, CFSE kit was purchased from Thermofisher (Waltham, MA, USA). Triton X100, sodium butyrate, sodium propionate, sodium acetate, Hoechst 33342, Laurdan, CTX-B-FITC, water soluble cholesterol (MBCD-cholesterol) was purchased from Sigma (St. Louis, MO, USA). Amplex red cholesterol assay kit, Trizol, Fillipin was purchased from Invitrogen (Carlsbad, CA, USA). Cholesterol, Cholesterol analogue (4-cholesten-3-one) and Phosphatidylcholine were obtained from Avanti polar lipids. Anti CD44-FITC antibody was purchased from BD Pharmigen^™^. Anti CD71-biotin was purchased from Bioss (Massachusetts, Boston, USA). Secondary Steptavidin PE was purchased from Biolegend (San Diego, CA, USA). Super Reverse Transcriptase MuLV Kit, RT [2] SYBR^®^ Green qPCR Mastermix, were purchased from Qiagen (Hilden, Germany). Protease inhibitor was purchased from Takara. Primers were purchased from IDT (Germany). RAW264.7 cell line was purchased from ATCC (USA).

### Propagation of RAW 264.7 cells, estimation of cellular protein and cholesterol

Murine macrophage cell line RAW264.7 were cultured in Dulbecco′s modified Eagle′s medium (DMEM) along with 10% Fetal Bovine Serum (FBS), supplemented with 1% Penicillin and Streptomycin at 37° C with 5% CO_2_ in the humified incubator (Heracell 150i, ThermoFisher Scientific). RAW 264.7 cells were seeded in 6 well tissue culture plates (10^6^ cells/ well) and allowed to adhere for overnight and reach confluency approximately 75–80%. Cells were then treated with different concentrations (5 mM or 10 mM) of sodium butyrate (butyrate) for 18 h having 2 mL media in each well. The viability and toxicity in the cells were assayed by Annexin/PI and LDH assay. Total crude cell membranes were isolated as described [24]. Cells were homogenized in 1 mL of buffer [10 mM Tris-HCl (pH 7.4), 1 mM EDTA, 200 mM sucrose] and protease inhibitor mix (Roche Diagnostics, Mannheim, Germany)]. The nuclei and cellular debris were removed by centrifugation at 900 × g for 10 min at 4 °C. The resulting supernatant was centrifuged at 100,000 × g for 75 min at 4 °C to obtain the crude membrane pellet. The pellet was resuspended in PBS and an aliquot of it was used for protein measurement using Pierce^™^ BCA Protein Assay Kit following manufacturer′s protocol. The rest of the pellet was extracted with 2∶1 methanol/chloroform, followed by 0.5 mL of chloroform and 0.5 mL of water. The methanol/chloroform (lipid phase) layer was dried under vacuum in a vacuum desiccator. The dry lipid was suspended in 200 µL of 1 × Reaction buffer supplied with Amplex Red Cholesterol Assay Kit and membrane cholesterol quantification was performed by following the manufacturer's instructions.

### Fillipin staining

Cells were washed with PBS twice, fixed with 2% chilled paraformaldehyde for 30 min at room temperature. After washing with PBS thrice the cells were incubated with 1 mL of 1.5 mg glycine/mL PBS for 10 min at room temperature to quench paraformaldehyde. Thereafter the cells were stained with 1 mL of 0.05 mg/mL of Fillipin in PBS containing 10% FBS for 2 h at room temperature. After washing with PBS thrice, the fluorescence images of the cells were visualized under Carl Zeiss microscope equipped with a CCD camera controlled with ZEN software (Carl Zeiss, Gottingen, Germany) and also analyzed using FACSDiva software in FACS Aria II machine (both Becton Dickinson, San Jose, CA). Each sample had at least 10,000 occurrences recorded in it. The data was analyzed using FloJo software.

### Membrane anisotropy

Membrane fluidity and fluorescence were measured as described [26, 52]. Briefly, Laurdan, the fluorescent probe, was dissolved in HPLC grade water to make a stock solution of 2 mM. This 1 mL stock solution was added to 10 mL of rapidly stirring PBS (pH 7.2). To 2 × 10^6^  cells in 1 mL PBS, 1 mL of Laurdan (C_*f*_ 1 µM) was added and incubated for 2 h at 37 °C. Following incubation, the cells were washed thrice and resuspended in PBS. The Laurdan probe bound to the membrane of the cell was excited at 350 nm and the intensity of emission was recorded at 435 nm in a spectrofluorometer (Cary Eclipse Instrument (MY13130004)). The fluorescence anisotropy (FA) value was calculated using the equation: FA = [(I_∥_ -I _⊥_)/ (I_∥_ + 2I _⊥_)], where I_∥_ and I _⊥_ are the fluorescent intensities oriented, respectively, parallel and perpendicular to the direction of polarization of the exciting light.

### Cholesterol-liposome and cholesterol analogue-liposome synthesis and treatment of liposomal cholesterol on cells

Liposomes were prepared by mixing cholesterol/ cholesterol analogue (4-cholesten-3-one) and Phosphatidylcholine according to the protocol [27]. Briefly, cholesterol/ cholesterol analogue (4-cholesten-3-one) and PC were mixed at a ratio of 1:1.5 in a round bottom flask and kept inside a vacuum desiccator for overnight. A thin film of lipid layer was obtained which was then dissolved in 1 mL DMEM and filtered with 0.22 µm of membrane filter. The size of the liposomes was measured by Differential light Scattering (DLS). Henceforth, cholesterol-liposome and analogue-liposome will be denoted as chol-lipo and ana-lipo respectively.

Freshly prepared 10 µL (containing approx 4.8 × 10^14^ lipoparticles) of liposomes/10^6^ cells were treated to sodium butyrate treated cells and incubated 18 h at 37 °C in 5% CO_2_ incubator. The concentration of liposome was determined by following equation (10.3390/scipharm89020015).

N_(lipo)_ = [M_(ing)_ x N_(Avo)_] / [N_(tot)_ × 1000].where:

N_(lipo)_ is the number of vesicles per mL;

M_(ing)_ is the molar concentration of ingredients of vesicles;

N_(Avo)_ is the Avogadro Number (6.02 X 10^23^);

N_(tot)_ is the total number of ingredients per vesicle.

N_(tot)_ is calculated using the following equation:

N_(tot)_ = 17.69 x [(d/2)100 + (d/2—5)100] where, d is the diameter of the vesicle.

### Visualization of liposomes in transmission electron microscopy (TEM)

To obtain a visual impression, the morphologies of liposomes were studied by performing TEM (200 kV FEG TEM) after negative staining of samples with uranyl acetate. To obtain information about lamellation, liposomes in PBS were mixed with an equal volume of 3% agar and kept at − 20 °C overnight. The solidified agar containing vesicles was cut into sections that were 60 nm thick with a cryo ultramicrotome. The sections were taken in a 300 mesh carbon coated Copper grid and dried overnight to observe in TEM.

### Confocal analysis and image processing

The cells were seeded on a 1.5H coverslip (0.16–0.19 mm thick) and treated with 10 mM sodium butyrate or 10 mM sodium butyrate followed by liposomal cholesterol. After 18 h the coverslips were washed with PBS after treatment and fixed by incubating the cells with 4% paraformaldehyde for 10 min at room temperature. Cells were washed with PBS for thrice and stained with CTXB-FITC or anti-CD71-biotin or anti-CD44-FITC antibody by incubating them for 30 min at 1:200 dilution. For CD71 detection, after 3 times PBS washing, the cells were further stained with streptavidin-PE antibody (1:200 dilution). The reaction was terminated by washing the cells thrice with PBS. Counter stain was done by treating the cells with 1 µg/mL Hoechst 33342 for 5 min at room temperature and washed again with PBS three times and mounted with 90% glycerol. Fluorescence images were captured in 63X magnification in confocal microscope (Carl Zeiss (Germany)). The analysis was done by ImageJ software. Corrected total cell fluorescence is calculated as:

Corrected Total cell fluorescence = (Integrated density)—(Area of selected cells x mean fluorescence of background).

### CD44 expression analysis by flowcytometry

To estimate the total expression of CD44 in both control and butyrate treated RAW 264.7 cells, the cells were permeabilised/ unpermeabilized using 0.1% Tween 20 for 15 min and then fixed with 2% chilled paraformaldehyde for 30 min at room temperature. Cells were then collected in FACS buffer (PBS + 10%FBS). Aliquotes containing 10^6^ cells in FACS buffer were stained for 30 min in room temperature using FITC conjugated anti-CD44 antibody in 1:500 dilutions. The cells were then examined using FACSDiva software in a FACS Aria II machine (both Becton Dickinson, San Jose, CA). Each sample had at least 10,000 occurrences recorded in it. The data was analyzed using FloJo software.

### Bacterial strain

*Shigella flexneri* serotype 2a strain 2457 T and *Salmonella* serovar typhimurium wild-type strain SL1344 were grown in Luria broth (LB) at 37 °C overnight and reinoculated with 1% precultured bacteria in fresh media in a shaking incubator at 37 °C. OD_600_ was measured by Spectrophotometer for monitoring and when the OD reached the value of 0.6, the culture was diluted to 2 × 10^9^ CFU/mL in PBS for further experiments.

### Pathogen invasion assay

Pathogen invasion assay [28] was performed in the cells cultured in serum free medium to avoid interference of serum cholesterol. Briefly, *Shigella flexneri* or *Salmonella typhimurium* was added to the cells (5 × 10^5^ cells/ well in 500 µL medium in a 24 well plate) at 100 multiplicity of infection (MOI) and incubated for 1 h. Media was aspirated and cells were washed thrice with PBS, followed by addition of DMEM containing 50 µg/mL of Gentamycin and incubated for 1 h at 5% CO_2_ at 37 °C. After incubation, media was removed and cells were washed thrice with PBS. Cell lysate was prepared by adding 100 µL of 0.1% Triton X-100 to the cells. The lysate was then plated on either XLD plates for *Shigella flexneri* or LB agar for *Salmonella typhimurium* and incubated at 37 °C overnight. The numbers of colonies were counted on the next day and the data is represented as percent control.

Percent control = Treated/Control × 100.

### Dietary supplementation of sodium butyrate and chol-lipo treatment in mice

The dietary supplementation studies were performed as reported earlier with minor modification [27]. Briefly, a group of 10 adult C57BL/6 mice (divided as 5 in each group) were fed with chow diet and 150 mM sodium butyrate in drinking water for 30 days. On 28th day of butyrate treatment, 5 animals from the group were selected randomly and was injected chol-lipo (200 µL of liposomal suspension) through intracardiac route (chol-lipo mice) [24]. Another set of 5 mice with regular chow diet and normal drinking water served as normal group.

These animals were infected intraperitoneally with 10^8^ cfu/mice of *Shigella flexneri* on 30th day. On day 2 post infection the animals were sacrificed and biomaterials were collected for further analysis. A group of age matched normal mice served as uninfected control.

### Bacteria count (CFU) from colon tissue

The colon tissue (40–50 mg) collected from mice were extensively washed with PBS, resuspended in 1 mL PBS and homogenised using Dounce Homogeniser. The tenfold diluted homogenate was plated in XLD agar plates and subjected to overnight incubation at 37 °C. Colonies were counted on the following day. The cfu/ gm tissue was calculated as.

Cfu/ gm tissue = (number of colonies x dilution factor)/gm of tissue.

### Histopathological analysis

Colon samples were washed thoroughly with PBS and fixed in 4% paraformaldehyde at 4 °C for 48 h and then dehydrated by soaking then in graded alcohol, xylene, embedded in paraffin. 5 µm sections were obtained from paraffin block by routine microtome and stained with H&E and subjected to microscopic analysis (Carl Zeiss Axiovert 40 CFL). The degree of interstitial infiltration by inflammatory cells, in response to *Shigella* infection, was evaluated by counting the number of neutrophils in the colon at high power field (X100) as previously described [29].

### RNA extraction and reverse transcription

The cytokine mRNA expression was studied from gut tissue as reported previously [30]. Total RNA from the tissue was extracted with Trizol according to the protocol recommended by the manufacturer. The concentration of the extracted RNA was analyzed by Nanodrop spectrophotometer (Thermo) and RNA was stored at –80° C. cDNA was prepared from total RNA by reverse specific primers using Super Reverse Transcriptase MuLV Kit. The primers for the reverse transcription are listed in Table 1. GAPDH was normalized for the expressions of each gene. The total reaction volume for reverse transcription was 20 μl in which 1 μM of reverse primer, 5 ng of RNA template, 1 μl dNTP mix, 12 μl of DEPC treated water, 4 μL of 5 X first strand buffer, 1 μL of 0.1 M DTT, 1 μL of RNase inhibitor and 1 μL Super RT MuLV. Reverse transcription was carried out for 65 °C for 5 min, followed by incubation at 55 °C for 1 h and then heat inactivating the reaction at 70 °C for 15 min.

**Table 1:** List of Primers used in the study

Gene (Accession number of the gene)	Primer sequence	
	Forward primer	Reverse primer
GAPDH (NM_008084.4)	5′-AGAGAGGCCCAGCTACTCG-3′ (Tm = 59.8)	5′GGCACTGCACAAGAAGATGC-3′ (Tm = 59.9)
MUC-2 (NM_023566.4)	5′-GCTGACGAGTGGTTGGTGAATG-3′ (Tm = 60.0)	5′- GATGAGGTGGCAGACAGGAGAC-3′ (Tm = 59.9)
IFN-γ (NM_008337.4)	5′-TCAAGTGGCATAGATGTGGAAGAA-3′ (Tm = 59.9)	5′-TGGCTCTGCAGGATTTTCATG-3′ (Tm = 59.9)
IL-10 (XM_036162094.1)	5′-GGTTGCCAAGCCTTATCGGA-3′ (Tm = 60.0)	5′-ACCTGCTCCACTGCCTTGCT-3′ (Tm = 59.9)
TNF-α (NM_001278601.1)	5′-CATCTTCTCAAAATTCGAGTGACAA-3′ (Tm = 60.1)	5′-TGGGAGTAGACAAGGTACAACCC-3′ (Tm = 60.2)
IL-6 (NM_001314054.1)	5′-GATAAGCTGGAGTCACAGAAGG-3′ (Tm = 59.3)	5′-TTGCCGAGTAGATCTCAAAGTG-3′ (Tm = 60.0)
IL-12 (NM_001303244.1)	5′-GGAAGCACGGCAGCAGAATA-3′ (Tm = 59.8)	5′-AACTTGAGGGAGAAGTAGGAATGG-3′ (Tm = 60.1)
Cathelicidin (NM_009921.2)	5′- GGCAGCTACCTGAGCAATGT-3′ (Tm59.6)	5′-CTGTGCACCAGGCTCGTTA-3′ (Tm = 59.8)

### Quantitative real-time PCR

Total RNA was extracted with Trizol reagent from snap frozen liver and RNA concentration was determined using a nanodrop spectrophotometer. The genes and GAPDH levels were quantified with Applied Biosystems^™^ StepOne^™^ Real Time PCR System with RT^2^ SYBR^®^ Green qPCR Mastermix following the manufacturer′s instructions. Each 20 μL qPCR reaction contained an amount of cDNA equivalent to 5 ng of total RNA, 10 μL of RT^2^ SYBR^®^ Green qPCR Mastermix, 1 μM of the forward and reverse primer (each) and nuclease free water. Real-time PCR was performed with the following conditions: 95 °C for 10 min, 40 cycles of 95 °C for 30 s, 60 °C for 1 min and 72 °C for 1 min PCR product was calculated according to the 2^^–ΔCt^ method described previously [31].

### Primers

The primer sequences have been designed by using NCBI Primer BLAST (https://www.ncbi.nlm.nih.gov/tools/primer-blast/). Following few things were considered for selecting the primers: 18–24 bases long, 40–60% G/C content, having 1–2 G/C pairs in beginning and end, 50–60 °C melting temperature (Tm) and primer did not have complimentary areas and their Tms were within 5 °C of one another. The list of primers use for PCR amplification is as follows in Table 1.

### Statistical analysis

All data are reported as means ± Standard Error Mean (SEM). All the data were reanalyzed with GraphPad Prism Version 8.01 software and statistical significance between more than two groups were determined by one way analysis of variance (ANOVA) followed by Tukey′s post hoc test. The p values of < 0.05 were considered statistically significant. The necessary changes were made under the section.

## Results

### Butyrate treatment decreases membrane cholesterol in RAW 264.7 cells

**Fig. 1:**
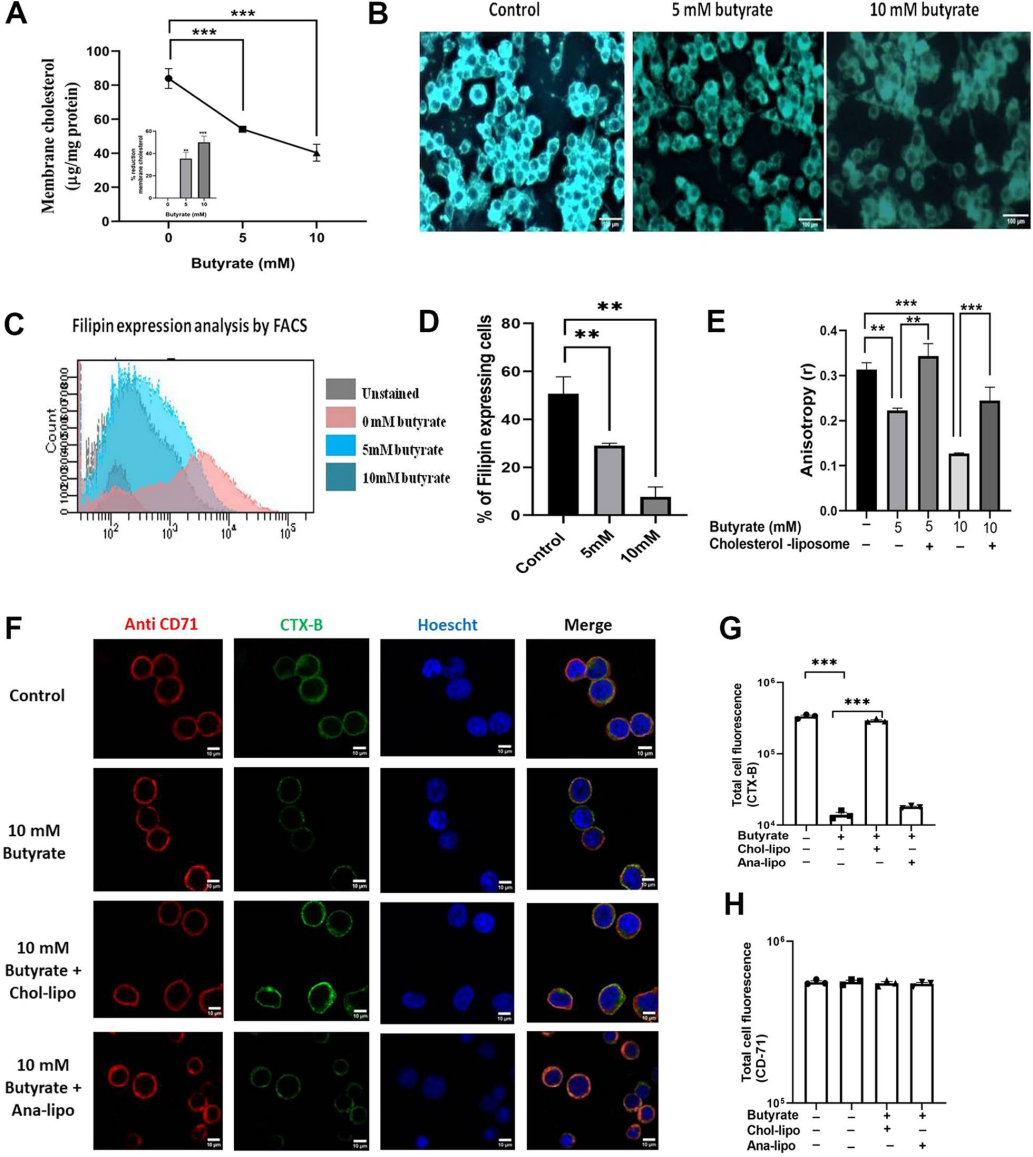
Butyrate treatment decreases membrane cholesterol, increases fluidity and disrupts lipid rafts in RAW264.7 cells. RAW 264.7 cells were treated with 5 mM or 10 mM or without sodium butyrate for 18 h. The cells were washed and membrane was prepared. The membrane cholesterol was estimated by amplex red cholesterol assay kit (invitrogen) and expressed as µg of membrane cholesterol/ mg protein **A**, the percent reduction of membrane cholesterol (Inset). The cells treated with/without butyrate were stained with Fillipin and observed under fluorescence microscope **B** and measured in flowcytometry **C**, **D**. After 18 h of butyrate treatment the cells were further treated with or without chol-lipo for another 18 h. The membrane anisotropy was measured by using Laudran probe and expressed as r **E**. The cells were stained with either CTX-B-FITC or anti-CD71 antibody and counterstained with Hoechst 33342. The cells were imaged in Zeiss confocal microscope **F**. The quantitative analysis of total fluorescence of CTX-B **G**, and anti-CD71 antibody **H**, as measured by ImageJ. The corrected total cell fluorescence is calculated as Corrected total fluorescence (CTCF) = (Integrated density)—(Area of selected cells x mean fluorescence of background). Each experiment were set in triplicate and the data of three experiments are plotted as Mean ± SEM. * represents p < 0.05, ** represents p < 0.01, *** represents p < 0.001

To assess the effects of butyrate on membrane cholesterol, RAW264.7 cells were treated with increasing concentration (5 mM and 10 mM) of sodium butyrate (butyrate) for 18 h following which membrane was prepared and cholesterol content was measured. Treatment with 5 mM and 10 mM of butyrate showed 1.5 fold and 2.4 fold decrease in membrane cholesterol (Fig. 1A) which corresponds to 36% and 52% decrease (Fig. 1A inset) with respect to control respectively. Staining with fillipin to detect cholesterol also showed decrease in fluorescence with butyrate treatment compared to control (Fig. 1B). Sodium butyrate treatment to RAW 264.7 neither decreased cell viability or confluency, proliferation nor did it induced toxicity in the cells even at the highest concentration that was used (Additional file 1: Fig S1). Butyrate treatment induced changes in the morphology of the cell which was evident from the microscopy images (Additional file 1: Fig S1D).

As membrane cholesterol consists of 60–80% of the total cellular cholesterol [32], its depletion is likely to change the physical properties of the membrane. To estimate the changes in the physical properties we next measured the membrane fluidity in butyrate treated and untreated cells.

### Butyrate treatment increases membrane fluidity and disrupts cholesterol rich microdomains

Fluidity of the cells treated with or without butyrate was measured by fluorescence anisotropy (FA) using Laurdan probe. It was observed that there was a dose dependent decrease in the FA value following treatment with butyrate. With 5 mM and 10 mM butyrate treatment FA was reduced to 1.4 fold and 2.4 fold respectively (Fig. 1E). To ensure the change in membrane fluidity was due to depletion of cholesterol we replenished cholesterol into the cell membrane of butyrate treated cells using liposomal delivery of cholesterol (chol-lipo). Liposomes were prepared using phosphatidyl choline (PC) with cholesterol. TEM confirmed that liposomes were indeed formed upon sonication (Additional file 1: Fig S2A). The Differential Light Scattering (DLS) study showed the liposomes prepared are of similar size distribution (Additional file 1: Fig S2B). When cholesterol was restored in the cells treated with butyrate by chol-lipo, the FA was reversed essentially to normal level (Fig. 1E). To warrant the increase in the membrane cholesterol with chol-lipo treatment we estimated the membrane cholesterol of the chol-lipo treated cells (Additional file 1: Fig S2C) using medium deficient in cholesterol (serum free condition). It was observed that approximately 16% and 40% cholesterol was restored in membranes upon chol-lipo treatment in 5 mM and 10 mM butyrate treated cells respectively.

For linking butyrate with decrease in membrane fluidity, we tested the presence of lipid microdomains in butyrate treated and untreated cells. Cholera toxin B (CTX-B) binds to GM1 ganglioside present in the cholesterol rich microdomains in the membrane [33] and is known as biomarkers for these domains. In Fig. 1F the first row shows the control cells without any treatment, second row shows the cells treated with 10 mM butyrate, the third row shows the cells treated with 10 mM butyrate followed by chol-lipo and the fourth row shows the cells treated with 10 mM butyrate followed by ana-lipo treatment. Column one depicts anti-CD71 binding, column 2 indicates CTX-B binding, column 3 shows the nucleus stain and column four is the merge image. The fluorescence images were quantified using imageJ which showed reduced CTX-B-FITC binding in at 10 mM butyrate treatment compared to control and was reversed back upon further treatment with chol-lipo but not with ana-lipo (Fig. 1G). But the binding of anti-CD71 antibody that binds to CD71 which is located in the non-raft region of the membrane [34] remained unaltered irrespective of butyrate, chol-lipo or ana-lipo treatment (Fig. 1H).

Having established that butyrate disrupts cholesterol rich microdomains in the membranes, we next asked the question of its impact on the enteric pathogen invasion which exploits these domains for host cell entry.

### Butyrate treatment decreases enteric pathogen invasion

**Fig. 2:**
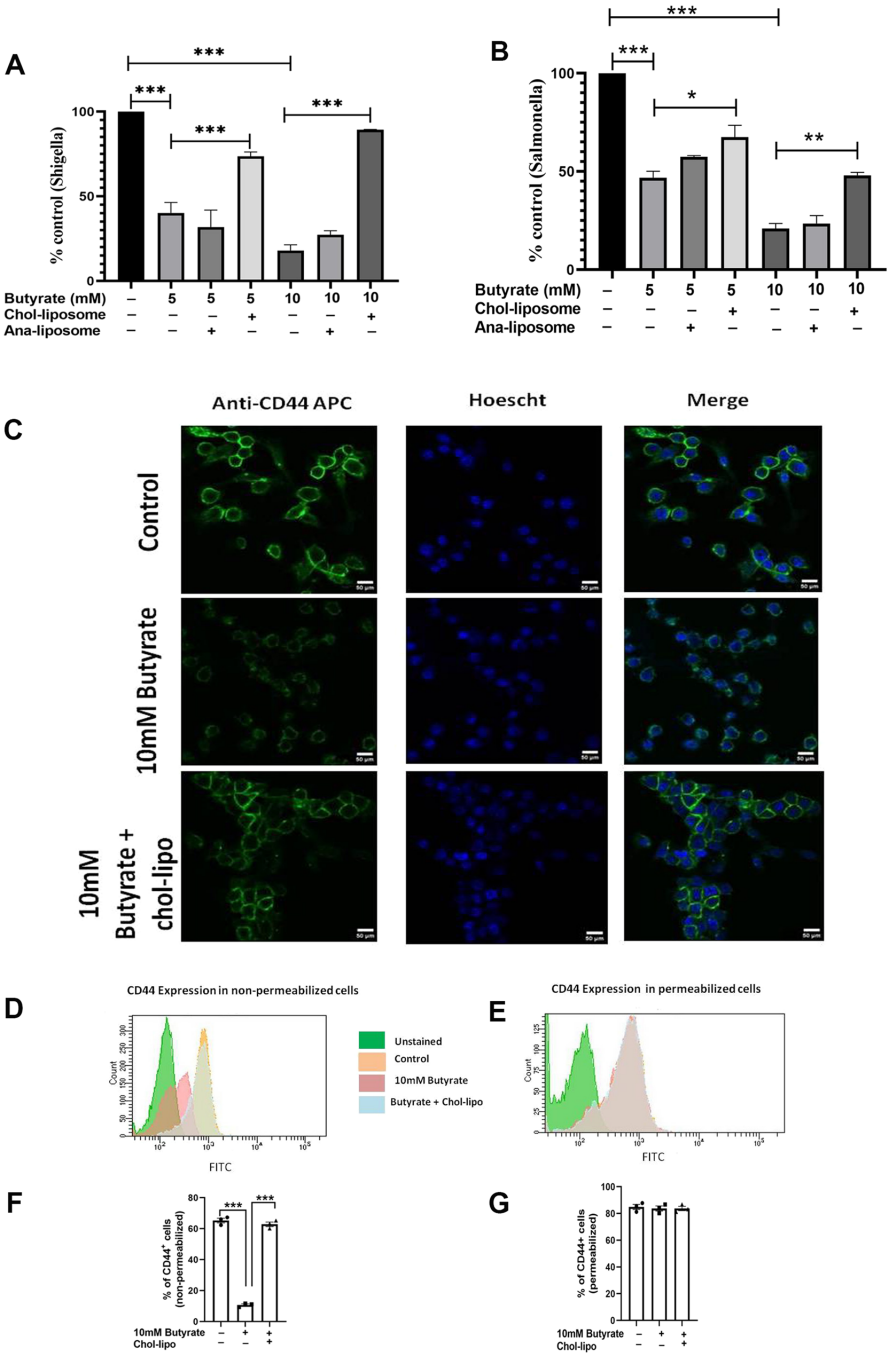
Butyrate treatment decreases pathogen invasion in macrophage cells. RAW264.7 cells were treated with or without butyrate and followed by chol-lipo was infected with *Shigella flexneri* **A** and *Salmonella typhimurium* **B** at a MOI 1:100 and the data are represented as percent control (Percent control = Treated/Control × 100). The cells were stained with either anti-CD44-FITC antibody and counterstained with Hoechst 33342. The cells were imaged in Zeiss confocal microscope at 63X magnification **C**. The cells with similar treatment were analyzed in flowcytometry in non-permeabilized **D** and permeabilized **E** to estimate the total fluorescence of anti-CD44-FITC antibody and on the cell surface respectively. The mean fluorescence was estimated and represented in F and G for non-permeabilized and permeabilized cells respectively. N = 3, the data is represented as Mean ± SEM. * represents p < 0.05, ** represents p < 0.01, *** represents p < 0.001

To study the effect of butyrate on enteric pathogen invasion we used *Salmonella typhimurium* and *Shigella flexneri* as representative enteric pathogens which exploits lipid rafts to enter the cell. The effect of butyrate treatment on the *Shigella* and *Salmonella* invasion in macrophages were analysed. It was observed that butyrate decreased the invasion of *Shigella* and *Salmonella* compared to infected control in a dose dependent manner. With 5 mM and 10 mM treatment of butyrate there was 60% and 85% decrease in *Shigella* invasion respectively and 60% and 75% decrease in *Salmonella* invasion respectively (Fig. 2A, B). Further treatment with chol-lipo to butyrate treated cells showed increased invasion of bacteria compared to butyrate treatment and essentially near to infected control for *Shigella* infection. To ensure the effect of reversal was due to cholesterol incorporation into the membranes and not due to enhanced phagocytosis caused by liposome treatment, we prepared liposomes using cholesterol analogue (4-cholesten-3-one) (ana-lipo) instead of cholesterol and applied to butyrate treated cells. As expected, the treatment with ana-lipo failed to increase the pathogen invasion and the percent of intracellular pathogens (*Shigella* and *Salmonella*) were near to butyrate treated macrophages.

Further studies with water soluble cholesterol or methyl β cyclodextrin (MBCD)-cholesterol (MBCD-chol) that transfer cholesterol from medium to cell membrane showed similar results. Replenishing membrane cholesterol by MBCD-chol in butyrate treated cells increased invasion of *Shigella* and *Salmonella* in a dose dependent manner (Additional file 1: Fig S3).

IpaB of *Shigella flexneri* binds to CD44 molecule [19] expressing on the surface of macrophage. Therefore, we checked the expression of CD44 with butyrate treatment.

### Butyrate treatment decreases anti-CD44 antibody binding on cell surface but does not change the total pool of CD44 in cell

The cells treated with butyrate shows decrease in anti-CD44-FITC antibody binding which was restored with further treatment with chol-lipo (Fig. 2C). To prove that the decrease in fluorescence was due to dislocation of CD44 from lipid rafts but not due to the decrease in overall cellular expression of CD44, we measured the expression of CD44 in permeabilized and unpermeabilized cells by flow cytometry. Permeabilization of cells enables the antibody to enter into the cell and bind to intracellular CD44, whereas in an unpermeabilized cell, the binding of the antibody occurs only on the cell surface wherever CD44 is expressed. Expression analysis of CD44 by flow cytometry on permeabilized cells clearly shows that the total expression of CD44 remains same irrespective of butyrate or chol-lipo treatment (Fig. 2E). But flowcytometry analysis of unpermeabilized cells showed reduced fluorescence with butyrate treatment which is restored with chol-lipo (Fig. 2F). This observation confirms that *Shigella* binding receptor CD44 localizes in the lipid rafts which are displaced after butyrate treatment.

To understand its ramification, we studied the effect of butyrate in *Shigella* infected mice.

### Butyrate treatment in mice reduces *Shigella flexneri* infection but reversed with liposomal delivery of cholesterol

**Fig. 3:**
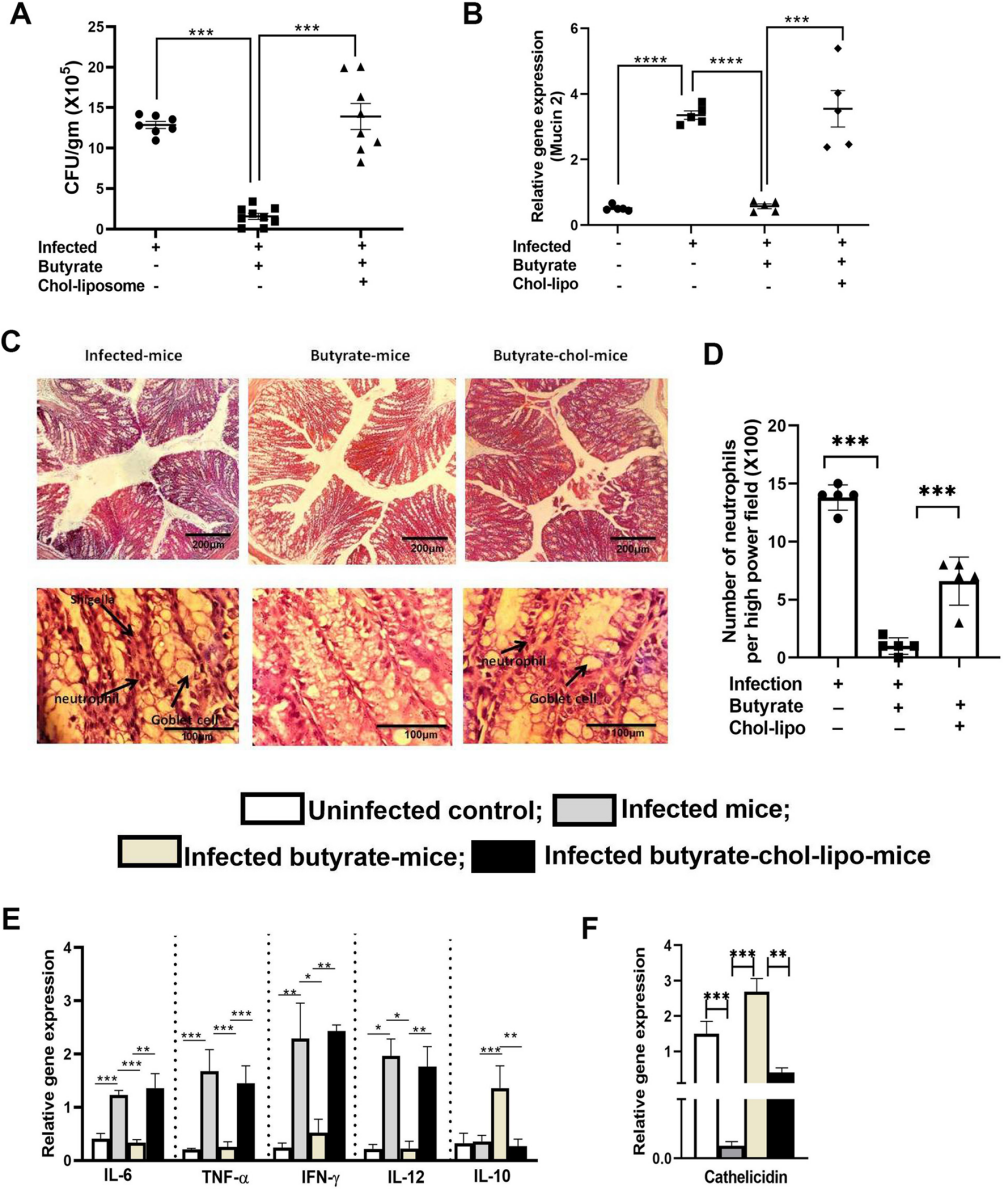
Butyrate treatment decreases pathogen invasion in mice which was reversed upon chol-lipo treatment. A group of adult C57BL/6 mice were fed with chow diet and 150 mM sodium butyrate in drinking water for 30 days. On 28^th^ day of butyrate treatment, half of the animals were selected randomly and was injected liposome-cholesterol (200 µL of liposomal suspension) through intracardiac route (chol-lipo mice). All the animals were infected with 10^8^ cfu/mice of *Shigella flexneri* on 30^th^ day intraperitoneally. On day 2 of post infection the animals were sacrificed and feaces and colon tissue were collected for analysis. The bacterial load was determined by plating diluted fecal samples on XLD plates. The bacterial load is determined as cfu/ gm feaces **A**. The mucin 2 expression was measured by qPCR **B**. The microscopic view of histological sections of the colon of representative animal per group **C**, number of neutrophils in high power field (100X) in the colon section of infected-mice, butyrate-mice and chol-lipo-mice **D**, Panel1, 10X magnification, Panel2, 100X magnification. The mRNA expression of the cytokines **E** and antimicrobial gene cathelicidin **F** from colon tissue was measured by qPCR. N = 3, 5 animals were taken per group, the data is represented as Mean ± SEM. * represents p < 0.05, ** represents p < 0.01, *** represents p < 0.001

Following 30 days of butyrate treatment, mice were infected with *Shigella flexneri* at a dose of 10^7^ cfu/ animal intraperitoneally and were sacrificed 24 h post infection to study pathogen burden and immune parameters. Hereafter the uninfected mice, *Shigella* infected mice and butyrate treated plus infected mice will be denoted as control-mice, infected-mice and butyrate-mice respectively. A group of butyrate-mice were further administered cholesterol liposome through intracardiac route 2 days prior to infection (butyrate-chol-mice). To ensure proper delivery of cholesterol in the gut, the cholesterol content of the gut tissue was measured (Additional file 1: Fig S4). It was observed that the cholesterol content in the gut tissue of infected-mice was significantly more than control-mice. As expected, the gut tissue cholesterol of butyrate-mice were decreased compared to infected-mice but was increased significantly in butyrate-chol-mice. Butyrate- mice showed nearly 12 fold decrease in bacterial load in the colon tissue compared to infected-mice. Further treatment with chol-lipo to butyrate-mice essentially restored bacterial load in the colon as compared to butyrate-mice (Fig. 3A).

### Butyrate treatment reduces *Shigella* induced pathogenesis in gut which was reversed by chol-lipo

The *Shigella* infected-mice showed pathological symptoms like excess secretion of mucin 2 in the colon that might originate from goblet cells. Butyrate-mice showed reduction in mucin 2 expression compared to infected-mice and in butyrate-chol-lipo-mice the mucin 2 expression was essentially equivalent to infected-mice (Fig. 3B). Of note, epithelial shedding, cell death in the crypt and villi, goblet cell hyperplasia and infiltration of inflammatory cells were found in the histological sections of the colon of infected-mice. Almost negligible number of neutrophils was infiltrated in the colon of butyrate-mice. Butyrate treatment did not change the gut pathology but in butyrate-chol-mice epithelial shedding, cell death was prominent (Fig. 3C). Chol-lipo treatment increased neutrophil infiltration significantly compared to butyrate-mice (Fig. 3D).

We found that proinflammatory cytokines such as IL-6, IFN-γ, and TNF-α were significantly higher in infected-mice than those of the control-mice (Fig. 3E). Butyrate treatment decreased the proinflammatory cytokines significantly which was restored to the equivalent level of infected-mice in butyrate-chol-mice. Further analysis of expression of host protective cytokine IL-10 shows 3.7 times increase in butyrate-mice compared to infected-mice. As expected, the expression of IL-10 decreased in butyrate-chol-mice and was essentially similar to infected-mice (Fig. 3E last column).

Further study on antimicrobial gene expression like cathelicidin showed significant increase in butyrate-mice compared to infected control and restored to infection level in butyrate-chol-mice (Fig. 3F).

## Discussion

We previously reported that butyrate but not propionate or aceatate reduces cholesterol synthesis following "AUF1-Dicer1-miR122" pathway [14]. In this discourse, we describe the host cell-dependent mechanism of gut microbial butyrate for resisting invasion of enteric pathogens. As macrophages are sentinel cells of immune homeostasis in gut [35] we undertook studies in RAW 264.7 as it is an appropriate model for macrophages [36] and the pathogens used in this study is reported to infect RAW 264.7 cells [37, 38]. The concentration of butyrate used in the study is in tune with the physiological concentration reported to be found in the intestine [39] and it did not show any change in cell proliferation, confluency and viability. We observed significant decrease in membrane cholesterol with butyrate treatment. Although butyrate treatment did not induce apoptosis in the cells, it altered the cell shape. Since cholesterol affects the mechanical properties of a cell through the underlying cytoskeleton [40], it is tempting to speculate that depletion of membrane cholesterol with butyrate treatment likely changes the cytoskeletal structure and therefore affects cellular morphology. Cholesterol plays a major role in determination of cell membrane properties as it regulates membrane fluidity [41]. We studied the macrophage membrane fluidity in terms of FA using Laurdan probe. By computing the generalised polarisation, alterations in the Laurdan emission spectrum caused by variations in membrane water content were quantified [42]. Employing this property Laurdan probe was shown to distinguish effects of perturbations on membrane fluidity by comparing the results of FA measurements [42]. The decrease in FA with butyrate treatment is an indicator of increase in the fluidity of the cell membrane which corresponds to the decrease in membrane cholesterol. Further treatment with chol-lipo to the butyrate treated cells reversed the fluidity of the membranes which is also correlated with restoration of membrane cholesterol. Chol-lipo composed of phosphatidyl choline and cholesterol at a ratio of 1:1.5 are unilamellar vesicles used in efficient delivery of cholesterol in the cells [43]. Reversal of FA with chol-lipo is indicative of the cholesterol depleting effect of butyrate from the cell membrane.

To link butyrate with decrease in membrane fluidity, we tested the presence of lipid microdomains or lipid rafts. Cholera toxin B (CTX-B) binds to GM1 ganglioside present in cholesterol rich microdomains on rafts [33]. Disruption of lipid rafts reduces CTX-B binding [44] and can be determined by decrease in fluorescence of CTX-B-FITC. Butyrate showed significant decrease in CTX-B binding compared to control and was reversed essentially to normal with chol-lipo treatment but not with ana-lipo treatment. On the other hand, anti CD71 antibody binding remained unaltered in all four cases. CD71 or transferrin receptors are located in non-raft region of the cell membrane and serves as a marker protein for non-raft region [45].

Pathogens exploit host lipid rafts to advance the infection [46]. Initial physical interaction of bacteria with a host cell is a key first step often determining what happens next, and there is no better place to gain control of the host cell than through initial contact with the lipid rafts. We have considered the invasion of enteric pathogens like: *Shigella* and *Salmonella* in our study. Replishment of cholesterol either by chol-lipo or MBCD-chol to butyrate treated cells restored pathogen invasion that was decreased with butyrate treatment. The initial steps of binding of the invasin IpaB of *Shigella flexneri* to CD44 present on the host cell membrane depends on the presence of cholesterol/shingolipid rich lipid domains [19]. *Salmonella* anchors to lipid raft associated CD55 [47] in cholesterol rich vacuoles [48]. We show that CD44 which was shown to be localized in membrane raft [49, 50] get internalized or relocate itself in non raft portion of the membrane in presence of butyrate without changing the overall expression. In conjunction to this, we provide evidence that butyrate treated cells significantly decreases invasion of both *Shigella* and *Salmonella* but with chol-lipo treatment the pathogen invasion reverts back to normal. Another report on a natural compound found in chillies called capsaicin which makes biomembranes fluid [51] is recently been shown to reduce *Shigella flexneri* infection in intestinal cells [52] adds further credence to our notion.

Since antibiotic treatment which is likely to disrupt gut microbiota and butyrate production in gut [53] is required prior to *Salmonella* infection in mice [54], we preferred to use *Shigella* infection (infected-mice) to validate our ex vivo results in mice model. Although there is no suitable animal model that can replace human-based studies for the investigation of the in vivo mechanisms of *Shigella* pathogenesis [55], a recent murine model based on peritoneal infection with virulent *S. flexneri* 2a is currently accepted [56]. In tune to the previous results, we observed significant decrease in bacterial burden in the colon of butyrate-mice compared to infected-mice. Supplementation of chol-lipo to butyrate-mice increased the pathogen burden compared to butyrate-mice. The half-life of the cholesterol rich unilamellar liposomes is longer than that of cholesterol poor liposomes in the body fluid [57]; Liposomes are believed to be targeted to liver and theoretically allow passage through large fenestrations, such as those of sinusoidal capillaries [58]; and while in circulation, liposomes readily absorb a vast collection of plasma proteins, some of which may act as opsonins directing liposomes to cell surface receptors and promoting their uptake by the cells [59]. We estimated the gut tissue cholesterol to ensure successful delivery of cholesterol into the gut. Notably infected mice also showed increase in colon tissue cholesterol. As *Shigella* infection causes decrease in butyrate producing bacteria in the gut [60], it is tempting to speculate that decrease in butyrate production increases cholesterol content in the gut of infected-mice. Similar increase in lipid profile was also noted in *Salmonella* infection which reverted back to normal with recovery [61]. Along with decreased bacterial load in gut, we also observed increase in mucin 2 expression, inflammatory cytokines, inflammatory cell infiltration and destruction of gut tissue lining. Previous studies suggest that mucin in the colon can be subject to direct colonic infection and inflammation [62, 63] For instance, Muc2^−/−^ mice exhibited rapid weight loss, high mortality, and greater bacterial burden when infected with *Citrobacter rodentium* [63]. Similarly, we find increase in mucin 2 expression in infected mice which was decreased significantly upon butyrate treatment and reversed in chol-lipo-mice. Thus, it seems likely that *Shigella* invasion and colonization of the colon can lead to multiple histological changes that are produced by the intrinsic host defense system in response to infection. The host defense systems against *Shigella* invasion were activated by secretion of cytokines and subsequent recruitment of immune cells to the site of infection [64].

Having established butyrate treatment reduces enteric pathogen invasion which can be reversed by liposome cholesterol we determined the host factors in the protection process. In order to further address whether butyrate treatment reduces the inflammatory responses in the colon elicited by *Shigella* infection, we determined secretion levels of cytokines expression in colon tissue. We found that proinflammatory cytokines such as IL-6, IFN-γ, and TNF-α were significantly higher in infected-mice than those of the control group. Butyrate treatment decreased the proinflammatory cytokines significantly which was restored to the equivalent level of infection group in butyrate-chol-mice. Collectively, these data indicate that the decrease in bacterial burden in butyrate-mice resident colon, *Shigella* organisms cannot provoke predominant cytokines, which contribute to lesser recruitment of polymononuclear cells into the colon. In turn, these may be involved in secretion of reduced proinflammatory cytokines. The antimicrobial protein cathelicidin is considered to play an important role in the defense mechanisms against bacterial infection in colonic macrophages [65]. Our results are in conjunction to earlier reports showing butyrate induces calthelicidin gene expression by augmenting its promoter activity [65].

Other than playing a role in pathogen invasion, lipid microdomains on the membrane plays crucial role in various essential cellular processes, including endocytosis, exocytosis and cellular signalling. Therefore, question arises whether butyrate treatment can disrupt any such process. It is evident that cholesterol depletion in the membrane causes spontaneous exocytosis [66]. Bearing with this knowledge butyrate also shows increased mucin secretion in the gut by exocytosis [67]. Membrane cholesterol is required for IFN-γ signalling [68]. Butyrate which we demonstrate as cholesterol decreasing agent is also reported to inhibit IFN-γ signalling [69]. Therefore, butyrate through a membrane centric enterprise shows gut homeostasis by making mucosal system being protective against pathogen invasion.

Overall, this discourse adds a new mechanism of colonization resistance in gut. We report that butyrate decreases membrane cholesterol which leads to increased fluidity and disruption of lipid microdomains which can be reversed by restoration of cholesterol in the cell membrane. Employing in vitro and in vivo experiments we have shown lipid raft disruption by butyrate decreases enteric pathogen invasion. By rescuing cholesterol in the membranes, we validate that butyrate treatment resist colonization of enteric pathogen by disruption of lipid microdomains in the membrane.

## Supplementary Information

**Additional file 1: Figure S1.** The viability, toxicity and proliferation of cells with/without butyrate treatment were measured by Apoptosis/PI staining in flowcytomestry (A), LDH assay (B) and CFSE staining in flowcytometry (C) respectively. The confluency of cells with/without butyrate treatment was observed under phase contrast microscope (magnification 20X) (D). The data is represented as Mean ± SEM of 3 independent experiments. * represents p<0.05, ** represents p<0.01. **Figure S2.** The shape (A) and size (B) of the chol-lipo and ana-lipo measured by TEM and DLS respectively. The cholesterol content of the membranes of the cells treated with/without butyrate followed by with/without chol-lipo was measured by amplex red cholesterol assay kit (C). The data is represented as Mean ± SEM of 3 independent experiments. * represents p<0.05, ** represents p<0.01, *** represents p<0.001. **Figure S3.** The cells treated with/without butyrate and then followed by MBCD-chol at an indicated concentrations were infected with *Shigella flexneri *(A) and *Salmonella typhimurium *(B) at a MOI 1:100 and the data are represented as percent control (Percent control = Treated/Control × 100). The data is represented as Mean ± SEM of 3 independent experiments. * represents p<0.05, ** represents p<0.01, *** represents p<0.001. **Figure S4.** The cholesterol content in the colon of normal, inf-mice, butyrate-mice and butyrate-chol-lipo-mice measured by Amplex red cholesterol assay kit. N = 5/group. The data is represented as Mean ± SEM of 2 independent experiments. ** represents p<0.01.

## Data Availability

The original contributions presented in the study are included in the article/supplementary material. Further inquiries can be directed to the corresponding author.

## Abbreviations

PBS: Phosphate buffered saline
IL: Interleukin
TNF: Tumour necrosis factor
IFN: Interferon
CD: Cluster of differentiation
qPCR: Quantitative polymerase chain reaction
Tm: Melting temperature
LDH: Lactate dehydrogenase
PI: Propidium iodide
GM1: Monosialotetrahexosylganglioside
Ipa: *Shigella* Virulence factor Invasion plasmid antigen
Sip: *Salmonella* Invasion protein
GAPDH: Glyceraldehyde-3-phosphate dehydrogenase
DEPC: Diethyl pyrocarbonate
DTT: Dithiothreitol
