# Supplementary material for: Butyrate driven raft disruption trots off enteric pathogen invasion: possible mechanism of colonization resistance
Source: Gut Pathog. 2023 Apr 21;15:19. doi: 10.1186/s13099-023-00545-0 (PMC10122309; doi:10.1186/s13099-023-00545-0)
Supplement: Supplementary file 1 — Additional file 1: Figure S1. The viability, toxicity and proliferation of cells with/without butyrate treatment were measured by Apoptosis/PI staining in flowcytomestry (A), LDH assay (B) and CFSE staining in flowcytometry (C) respectively. The confluency of cells with/without butyrate treatment was observed under phase contrast microscope (magnification 20X) (D). The data is represented as Mean ± SEM of 3 independent experiments. * represents p<0.05, ** represents p<0.01. Figure S2. The shape (A) and size (B) of the chol-lipo and ana-lipo measured by TEM and DLS respectively. The cholesterol content of the membranes of the cells treated with/without butyrate followed by with/without chol-lipo was measured by amplex red cholesterol assay kit (C). The data is represented as Mean ± SEM of 3 independent experiments. * represents p<0.05, ** represents p<0.01, *** represents p<0.001. Figure S3. The cells treated with/without butyrate and then followed by MBCD-chol at an indicated concentrations were infected with Shigella flexneri (A) and Salmonella typhimurium (B) at a MOI 1:100 and the data are represented as percent control (Percent control = Treated/Control × 100). The data is represented as Mean ± SEM of 3 independent experiments. * represents p<0.05, ** represents p<0.01, *** represents p<0.001. Figure S4. The cholesterol content in the colon of normal, inf-mice, butyrate-mice and butyrate-chol-lipo-mice measured by Amplex red cholesterol assay kit. N = 5/group. The data is represented as Mean ± SEM of 2 independent experiments. ** represents p<0.01. [file 13099_2023_545_MOESM1_ESM.pdf]

Butyrate driven raft disruption trots off enteric pathogen invasion: possible mechanism of colonization resistance

Running title: Butyrate disrupts lipid rafts and prevents pathogen invasion.

Oishika Das[1], Aaheli Masid[1], Mainak Chakraborty[1], Animesh Gope[1], Shanta Dutta[1] and Moumita Bhaumik[1*]

Fig S1:

The viability, toxicity and proliferation of cells with/without butyrate treatment were measured by Apoptosis/PI staining in flowcytomestry (A), LDH assay (B) and CFSE staining in flowcytometry (C) respectively. The confluency of cells with/without butyrate treatment was observed under phase contrast microscope (magnification 20X). The data is represented as Mean ± SEM of 3 independent experiments. * represents $p<0.05$, ** represents $p<0.01$

Fig S2

The shape (A) and size (B) of the chol-lipo and ana-lipo measured by TEM and DLS respectively. The cholesterol content of the membranes of the cells treated with/without butyrate followed by with/without chol-lipo was measured by amplex red cholesterol assay kit (C). The data is represented as Mean ± SEM of 3 independent experiments. * represents $p<0.05$, ** represents $p<0.01$, *** represents $p<0.001$

Fig S3

The cells treated with/without butyrate and then followed by MBCD-chol at an indicated concentrations were infected with *Shigella flexneri* (A) and *Salmonella typhimurium* (B) at a MOI 1:100 and the data are represented as percent control (Percent control= Treated/Control × 100). The data is represented as Mean ± SEM of 3 independent experiments. * represents $p<0.05$, ** represents $p<0.01$, *** represents $p<0.001$

Fig S4:

The cholesterol content in the colon of normal, inf-mice, butyrate-mice and butyrate-chol-lipo-mice measured by Amplex red cholesterol assay kit. N=5/group. The data is represented as Mean ± SEM of 2 independent experiments. ** represents $p<0.01$

Figure S1

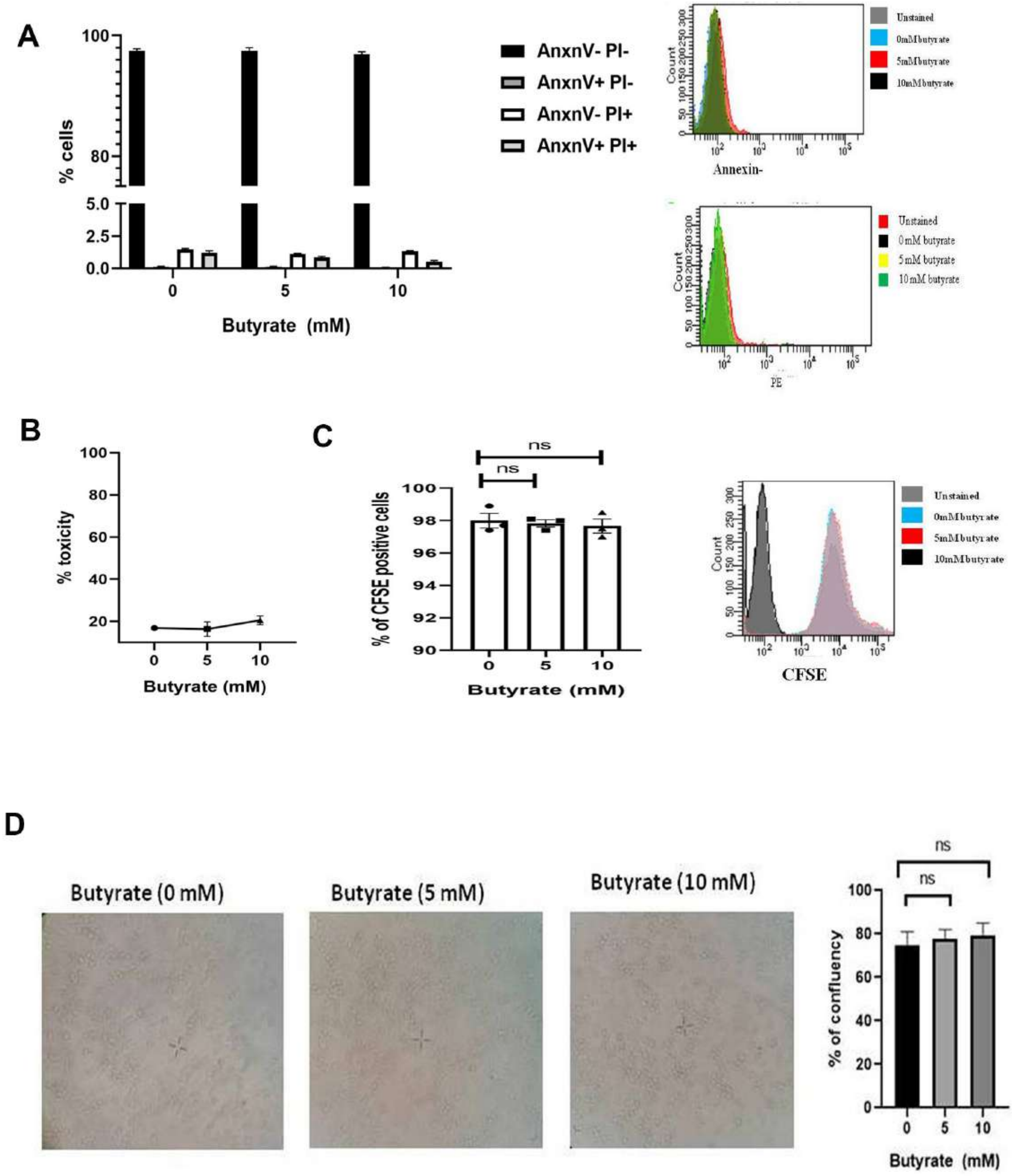

A
% cells
100
80
5.0
2.5
0.0
0
5
10
Butyrate (mM)
AnxnV- PI-
AnxnV+ PI-
AnxnV- PI+
AnxnV+ PI+
Count
Annexin-
Unstained
0mMbutyrate
5mMbutyrate
10mMbutyrate
PE
0 mM butyrate
5 mM butyrate
10 mM butyrate
B
% toxicity
Butyrate (mM)
C
ns
% of CFSE positive cells
Butyrate (mM)
CFSE
D
Butyrate (0 mM)
Butyrate (5 mM)
Butyrate (10 mM)
% of confluency
Butyrate (mM)

Figure S2

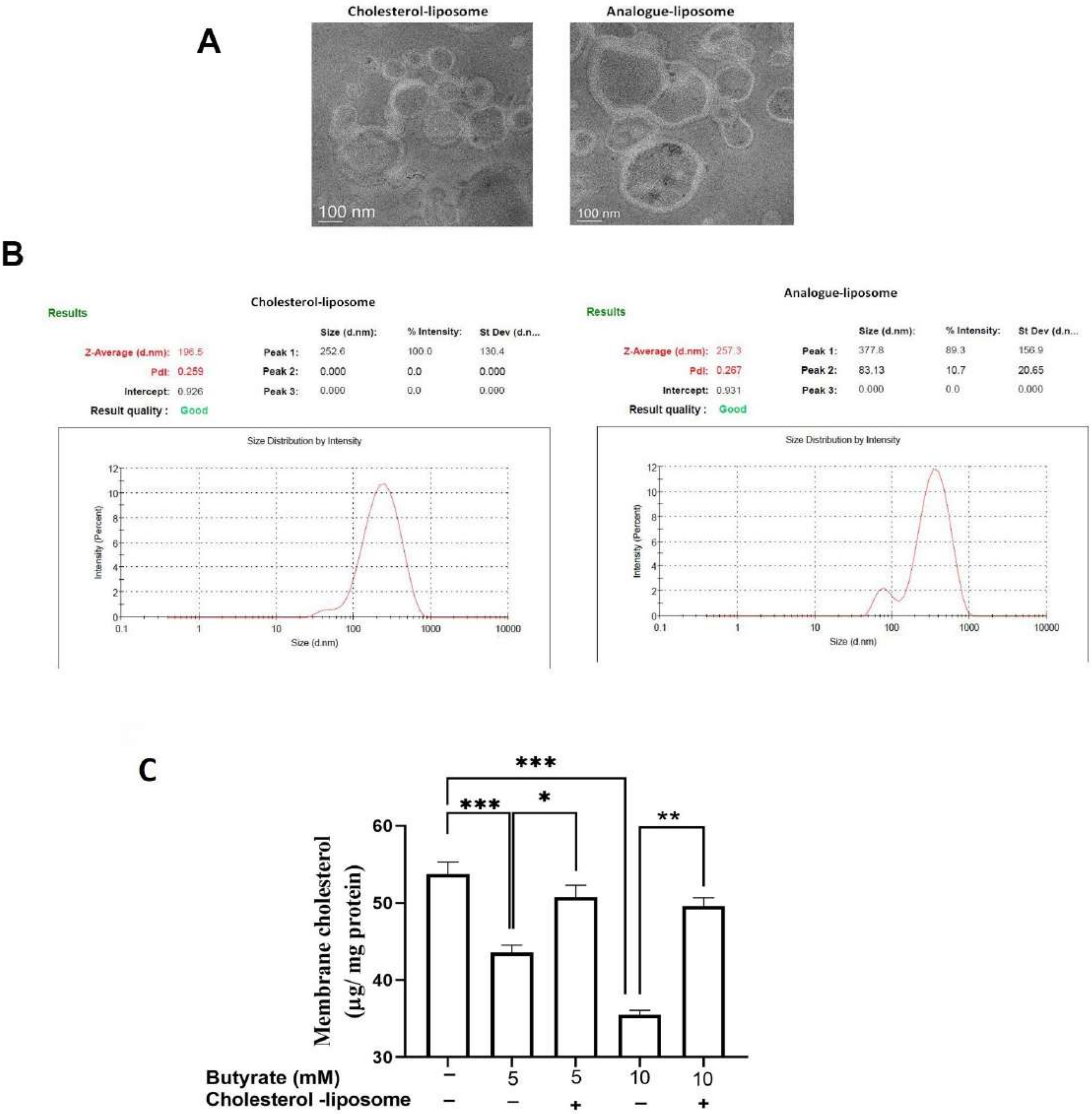
A
Cholesterol-liposome
Analogue-liposome
100 nm
100 nm
B
Cholesterol-liposome
Results
Size (d.nm): % Intensity: St Dev (d.n...
Z-Average (d.nm): 196.5 Peak 1: 252.6 100.0 130.4
PdI: 0.259 Peak 2: 0.000 0.0 0.000
Intercept: 0.926 Peak 3: 0.000 0.0 0.000
Result quality : Good
Size Distribution by Intensity
Intensity (Percent)
Size (d.nm)
Analogue-liposome
Results
Size (d.nm): % Intensity: St Dev (d.n...
Z-Average (d.nm): 257.3 Peak 1: 377.8 89.3 156.9
PdI: 0.267 Peak 2: 83.13 10.7 20.65
Intercept: 0.931 Peak 3: 0.000 0.0 0.000
Result quality : Good
Size Distribution by Intensity
Intensity (Percent)
Size (d.nm)
C
Membrane cholesterol (μg/ mg protein)
***
***
*
**
Butyrate (mM) – 5 5 10 10
Cholesterol -liposome – – + – +

Figure S3

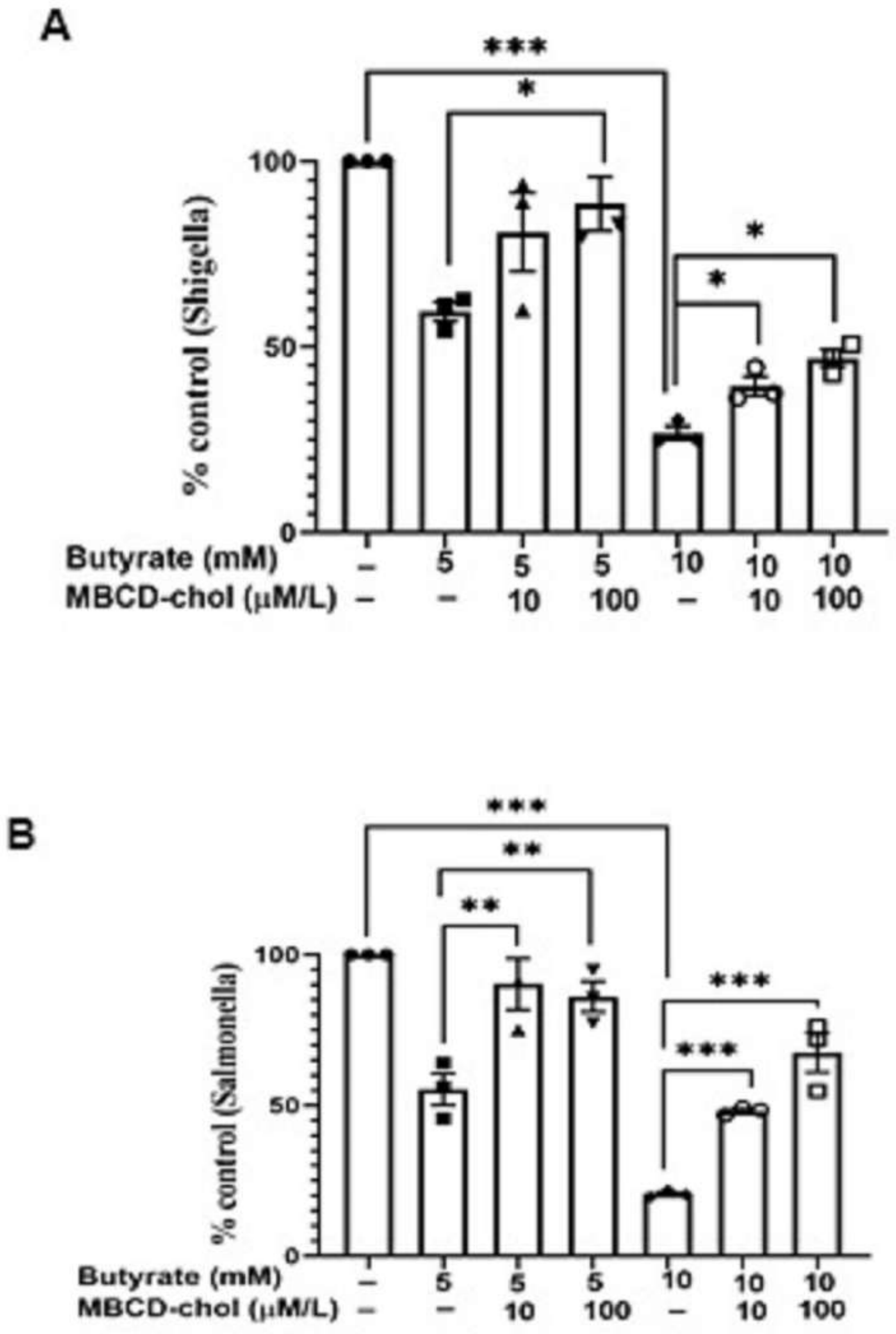
A
***
*
*
*
100
50
0
% control (Shigella)
Butyrate (mM) – 5 5 5 10 10 10
MBCD-chol (µM/L) – – 10 100 – 10 100
B
***
**
**
***
***
100
50
0
% control (Salmonella)
Butyrate (mM) – 5 5 5 10 10 10
MBCD-chol (µM/L) – – 10 100 – 10 100

Figure S4

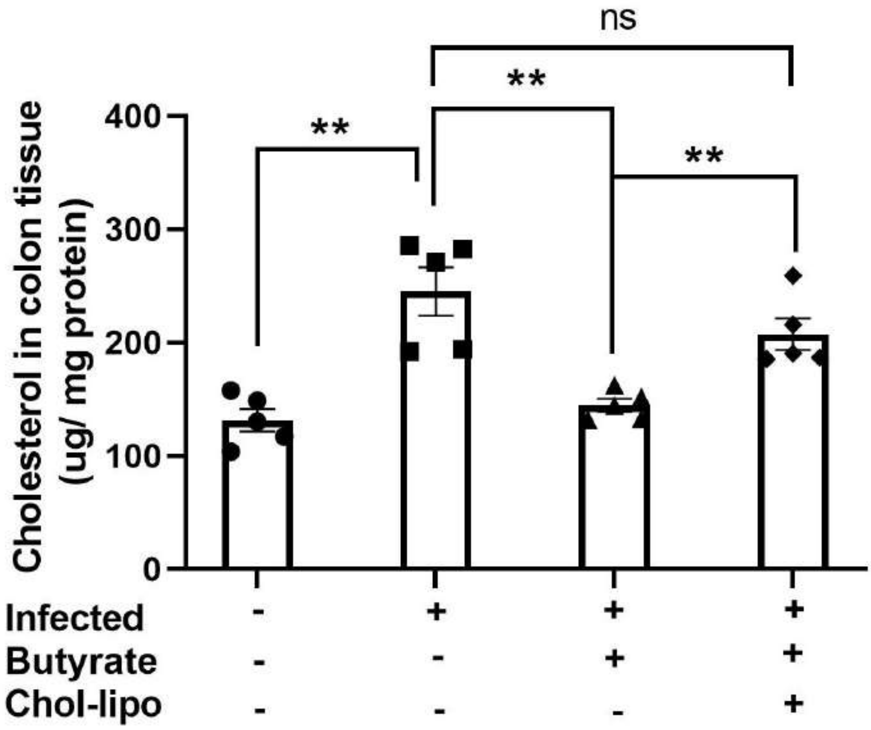
ns
**
**
**
Cholesterol in colon tissue
(ug/ mg protein)
400
300
200
100
0
Infected
Butyrate
Chol-lipo
- + + +
- - + +
- - - +
